# Metabolic activity of extranodal NK/T cell lymphoma on ^18^F-FDG PET/CT according to immune subtyping

**DOI:** 10.1038/s41598-021-85332-0

**Published:** 2021-03-15

**Authors:** Chae Hong Lim, Sang Eun Yoon, Seok Jin Kim, Junhun Cho, Young Hyeh Ko, Kyung-Han Lee, Won Seog Kim

**Affiliations:** 1 Department of Nuclear Medicine, Soonchunhyang University Hospital, Seoul, Korea; 2 Division of Hematology and Oncology, Department of Medicine, Samsung Medical Center, Sungkyunkwan University School of Medicine, 81, Irwon-ro, Gangnam-Gu, Seoul, 06351 Korea; 3 Department of Pathology, Samsung Medical Center, Sungkyunkwan University School of Medicine, Seoul, Korea; 4 Department of Nuclear Medicine, Samsung Medical Center, Sungkyunkwan University School of Medicine, 81 Irwon-ro, Gangnam-gu, Seoul, 06351 Korea

**Keywords:** Cancer, Immunology, Oncology

## Abstract

Disseminated extranodal NK/T cell lymphoma (ENKTL) is associated with dismal prognosis. Hence, distinct tumor immune microenvironment (TIME) subtypes were proposed to explain their influence on ENKTL progression and help predict treatment response. In this study, we investigated the capacity of FDG PET/CT to discern ENKTL TIME subtypes. A total of 108 pretreatment FDG PET/CT scans of 103 patients with newly diagnosed or relapsed ENKTL were retrospectively analyzed. TIME subtype was determined using three key immunohistochemical markers. SUVmax, MTV and TLG were measured, and metabolic features associated with TIME subtype were statistically extracted. TIME subtype was immune tolerance (IT) in 13.9%, immune evasion A (IE-A) in 56.5%, immune evasion B (IE-B) in 21.3%, and immune silenced (IS) in 8%. The IS group showed the highest SUVmax (15.9 ± 6.4, *P* = 0.037), followed by IE-A (14.1 ± 7.8), IE-B (10.9 ± 5.6), and IT groups (9.6 ± 5.1). Among 53 with only nasal FDG lesions, 52 had non-IS subtype. Among 55 with extra-nasal FDG lesions, those with IS subtype more often had adrenal (*P* = 0.001) or testis involvement (*P* = 0.043), greater MTV (*P* = 0.005), greater TLG (*P* = 0.005), and SUVmax located at extra-nasal sites. The presence of 0–2 and 3–4 of these four findings was associated with low probability (2/46) and high probability (6/9) of IS subtype, respectively. Furthermore, patients showing IS subtype-favoring PET/CT pattern had worse overall survival compared to their counterparts. These results demonstrate that FDG PET/CT can help predict immune subtype in ENKTL patients. The different patterns between glycolytic activity and involved site according to TIME subtype might be related to the interplay between tumor cells and immune cells in the tumor microenvironment.

## Introduction

Extranodal NK/T cell lymphoma (ENKTL), nasal type, is a rare type of lymphoma that ordinarily involves midline areas of the nasal cavity, oral cavity, and adjacent sites^1^. Some patients with ENKTL demonstrate distant nodal or extranodal involvement, which is associated with significantly worse survival outcomes^2,3^. At present, however, there is limited explanation as to why ENKTL cells migrate out of the nasal cavity in some cases.

Members of our group recently explored tumor immune microenvironment (TIME) biomarkers based on immunohistochemistry (IHC) and gene expression techniques that could help select patients with ENKTL that are more likely to respond to immunotherapy^4^. The work led to four distinct TIME subtypes based on expression of *FoxP3, PD-L1* and *CD68* markers: immune tolerance (IT), immune evasion A (IE-A), immune evasion B (IE-B), and immune silenced (IS) subtypes. Interestingly, the IT group, classified by high Treg count, was associated with an earlier stage and better survival outcome compared to other groups. Conversely, the IS group, classified by low *FoxP3* and *PD-L1* without high *CD68* (a unique morphologic change in CD68 and macrophages), displayed an exhausted immune response. Patients in this group mainly showed advanced stages and were resistant to immune checkpoint inhibitors, such as pembrolizumab and nivolumab^4^.

^18^F-fluorodeoxyglucose positron emission tomography/computed tomography (FDG PET/CT) is highly effective for detecting ENKTL lesions^5,6^. Furthermore, quantitation of FDG uptake in ENKTL lesions as maximum standard uptake value (SUVmax), metabolic tumor volume (MTV), or total lesion glycolysis (TLG) can provide important information regarding treatment response and prognosis^7–9^. Given the major role of tumor microenvironment in the metabolic activity of tumor cells and tumor-infiltrating immune cells^10^, metabolic parameters obtained from FDG PET/CT are likely to be influenced by the TIME.

Several studies describe the complex interactions between the immune environment and cancer cells and their influence on tumor development and metastasis^11,12^. However, it remains unknown how FDG uptake pattern might be associated with the state of the immune environment surrounding ENKTLs according to histologic and clinical data. This study was conducted to investigate PET/CT manifestations that help to characterize the TIME in ENKTL. Furthermore, the study was organized to determine whether IS subtype, which has poor response to immunotherapies, might be distinguished by FDG PET/CT.

## Materials and methods

### Patients

Data from 111 patients diagnosed with ENKTL at our institution who underwent FDG PET/CT within 30 days of formalin-fixed paraffin-embedded tumor biopsy between 2000 and 2017 were retrieved. Among these candidates, we excluded three patients who had excisional biopsy or surgical resection of the tumor before PET/CT was performed and five patients with tumor tissue acquired from unresolved lesions during chemotherapy. In addition, five patients who experienced relapse and showed different TIME subtypes for the first and second biopsies were also excluded. Thus, a total of 108 TIME cases were finally evaluated for analysis.

Clinical information was retrospectively obtained from electronic medical records to include clinical characteristics of age, sex, serum EBV DNA copy number, Ann Arbor stage, and prognostic index of natural killer lymphoma (-Epstein-Barr virus) (PINK(-E)). This study was approved by Samsung Medical Center institutional review board (IRB 2016–11-040–016) in accordance with the tenets of the Declaration of Helsinki^13^. All patients provided written informed consent.

### Tumor immune microenvironment (TIME) subtyping

A total of 108 tumor tissue sections was reviewed, comprising 103 samples obtained at initial diagnosis and five samples obtained at disease relapse. As previously described, the TIME subtype was categorized using three immunohistochemical markers, *FoxP3, PD-L1,* and *CD68*^4^. The four TIME subtypes identified were immune tolerance (IT), immune evasion A (IE-A), immune evasion B (IE-B), and immune silenced (IS).

### FDG PET/CT imaging

The PET/CT protocol of our institution was previously described in detail^14^. All patients fasted for at least six hours, and blood glucose was less than 150 mg at the time of PET/CT. Imaging was performed 60 min after injection of 5 MBq/kg of FDG without intravenous or oral contrast on a Discovery LS (GE Healthcare, Chicago, IL, USA) or a Discovery STe PET/CT scanner (GE Healthcare, Chicago, IL, USA). Continuous spiral CT was performed with an eight-slice helical CT (140 keV; 40–120 mA; Discovery LS) or 16-slice helical CT (140 keV; 30–170 mA; Discovery STe). An emission scan was then obtained from head to thigh or foot for 4 min per frame in two-dimensional mode with reconstruction of attenuation-corrected PET images (4.3 × 4.3 × 3.9 mm). Reconstruction was conducted using an ordered-subset expectation–maximization algorithm (28 subsets, two iterations: Discovery LS). Alternatively, 2.5 min per frame in three-dimensional (3D) mode with reconstruction of attenuation-corrected PET images (3.9 × 3.9 × 3.3 mm) were used with a 3D ordered-subset expectation–maximization algorithm (20 subsets, two iterations: Discovery STe).

### Review of PET images and analysis of FDG uptake

A board-certified nuclear medicine physician analyzed FDG PET/CT images using MIM version 6.4 software (MIM Software Inc., Cleveland, OH). ENKTL-involved FDG lesions were defined as lesions with focal FDG uptake greater than surrounding tissues unrelated to normal physiologic or benign activity. Abnormal FDG-avid sites in the upper airway tract including nasopharynx and paranasal sinus were categorized as nasal lesions. Any abnormal FDG-avid site outside this region was classified as an extra-nasal lesion. Quantification of FDG uptake as SUVmax, MTV, and TLG was measured in the involved lesion containing the area of highest visual activity. The tumor edge was delineated using a gradient-based segmentation method that exploits the gradient between high SUV of tumor cells and lower SUV of adjacent tissues^15^. The physician selected the image slice in which the target tumor appear largest. The physician then localized a point near the center of the lesion in this slice and dragged the cursor out to a point near the edge of the lesion. Six axes interactively extended out and the length of an axis was restricted when a large gradient was detected along that axis. After releasing the mouse button, the software automatically outlined a three-dimensional volume of interest (VOI) on the tumor^16^. TLG was calculated as the product of SUVmean and MTV.

### Statistical analyses

Differences in variables according to immune subtype were compared by the Kruskal–Wallis test with Dunn post-hoc tests (continuous variables) and chi-square tests (categorical variables). Differences in continuous variables between IS and non-IS types were assessed using Mann–Whitney *U* tests. Receiver operating characteristics (ROC) curve analysis was performed to identify optimal cutoffs of FDG parameters for predicting IS subtype. Kaplan–Meier curve (log-rank test) was used for survival analysis. All statistical tests were two-sided with a significance level set at 0.05 and performed with the Statistical Package for Social Sciences version 23.0 (IBM Corp., Armonk, NY, USA) and MedCalc version 15.5 (MedCalc, Mariakerke, Belgium).

## Results

### Clinical and pathological features

Baseline demographic and clinical characteristics of the 103 study subjects are summarized in Table 1. Twenty-eight patients were older than 60 years, and the male to female ratio was 2.2:1. Forty-seven patients (45.6%) with serum EBV detection and 43 patients (41.8%) with high PINK-E score were included. When the cohort was classified into TIME subgroups at diagnosis, IT, IE-A, IE-B, and IS groups comprised 15 cases (14.6%), 59 cases (57.3%), 21 cases (20.4%), and 8 cases (7.8%), respectively. In addition, two cases of IE-A, two cases of IE-B, and a case of IS in which TIME subtype was confirmed at relapse were also included for analysis.

**Table 1:** Patient characteristics.

Variables		N (%)	
**At diagnosis (N = 103)**			
Sex	Male	71	68.9
	Female	32	31.1
Age	< 60	75	72.8
	≥ 60	28	27.2
LDH	Elevated	51	49.5
	Not elevated	52	50.5
Serum EBV	Detected	47	45.6
	Not detected	46	44.7
Ann Arbor stage	I-II	70	68.0
	III-IV	33	32.0
PINK	Low risk	22	21.4
	Intermediate risk	32	31.1
	High risk	49	47.5
PINK-E	Low risk	47	45.6
	Intermediate risk	13	12.6
	High risk	43	41.8
TIME subgroup	IT	15	14.6
	IE-A	59	57.3
	IE-B	21	20.4
	IS	8	7.8
**At relapse (N = 5)**			
TIME subgroup	IT	0	0
	IE-A	2	40
	IE-B	2	40
	IS	1	20

LDH, lactate dehydrogenase; EBV, Epstein-Barr virus; LN, lymph node; PINK, prognostic index for extranodal NK/T-cell lymphoma; CTx, chemotherapy; RTx, radiotherapy; HSCT, hematopoietic stem cell transplantation; TIME, tumor immune microenvironment; IT, immune tolerance; IE, immune evasion; IS, immune silenced.

### PET/CT pattern according to immune subtype in the entire cohort

In the entire cohort (N = 108), IT, IE-A, IE-B, and IS groups numbered 15 (13.9%), 61 (56.5%), 23 (21.3%), and 9 cases (8.3%), respectively. IT and IE groups occurred more frequently at first diagnosis, whereas the IS group was more common in relapsed cases. PET/CT findings and metabolic parameters according to immune subtype are listed in Table 2. Subjects showing extra-nasal FDG lesions were more likely IE-B or IS groups. The IT group mainly showed FDG lesions restricted to the nasal cavity. When metabolic activity was compared, tumors of the IS subtype had the highest SUVmax (mean ± SD: 15.9 ± 6.4), followed by IE-A (14.1 ± 7.8), IE-B (10.9 ± 5.6), and IT subtypes (9.6 ± 5.1).

**Table 2:** FDG PET/CT pattern according to immune subtype.

	Immune subtype				
	IT (N = 15)	IE-A (N = 61)	IE-B (N = 23)	IS (N = 9)	*P*-value
**Diagnosis type**					
Initial diagnosis	9 (60%)	48 (79%)	19 (83%)	4 (44%)	0.066†
Relapse	6 (40%)	13 (21%)	4 (17%)	5 (56%)	
**PET/CT pattern**					
Nasal only	10 (67%)	33 (54%)	9 (39%)	1 (11%)	0.036†
Extranasal only	3 (20%)	9 (15%)	1 (4%)	4 (44%)	0.044†
Nasal + extranasal	2 (13%)	19 (31%)	13 (57%)	4 (44%)	0.037†
**SUVmax**					
Median (25% ~ 75%)	8.7 (6.7–12.1)	12.8 (9.5–16.5)	9.9 (7.2–15.4)	17.2 (11.5–20.4)	0.037‡
Mean ± SD	9.6 ± 5.1	14.1 ± 7.8	10.9 ± 5.6	15.9 ± 6.4	

IT, immune tolerance; IE, immune evasion; IS, immune silenced; SUV, maximum standardized uptake value; SD, standard deviation; †, Chi-squared test ; ‡ Kruskall-Wallis test.

### Metabolic parameters in the sub-population with extra-nasal FDG lesions

**Figure 1:**
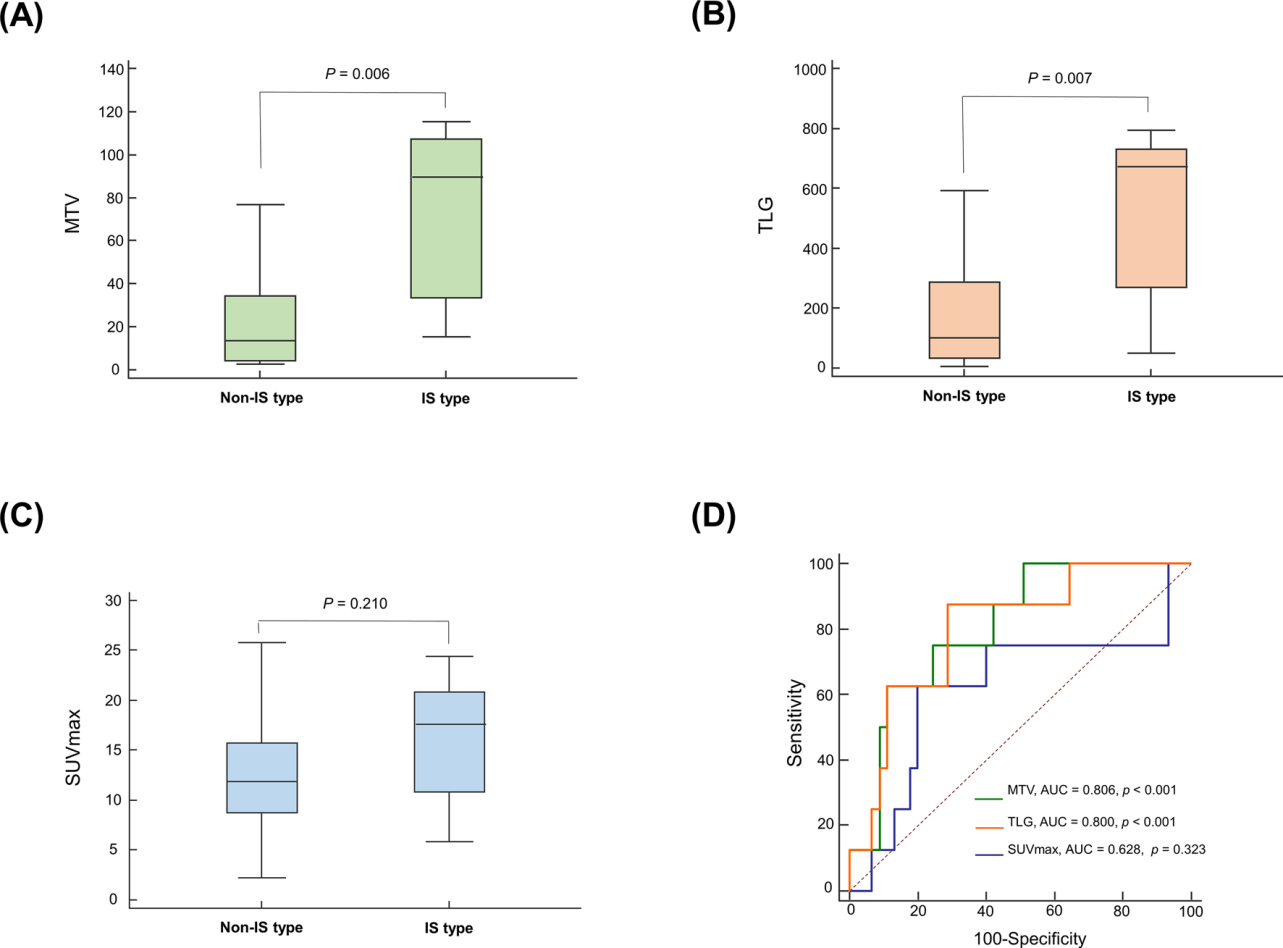
Box-whisker plots for metabolic tumor volume (MTV; **A**), total lesion glycolysis (TLG; **B**), and SUVmax (**C**) between non-IS and IS TIME subtypes in ENKTL patients with extra-nasal involvement. (**D**) ROC curves illustrating performance of SUVmax, MTV, and TLG for differentiating IS from non-IS subtypes. IS, immune silenced.

Fifty-five cases demonstrated FDG lesions outside the upper airway tract. This included 5 cases of IT, 28 cases of IE-A, 14 cases of IE-B, and 8 cases of IS. The IS type had significantly greater MTV (Fig. 1A, *P* = 0.006) and TLG (Fig. 1B, *P* = 0.007) compared to non-IS types. SUVmax showed a similar trend, although not to a statistically significant degree (Fig. 1C; *P* = 0.210). ROC analysis demonstrated that MTV (AUC = 0.806; *P* =  < 0.001) and TLG (AUC = 0.800; *P* =  < 0.001) were significant predictors for IS subtype (Fig. 1D; optimum cutoffs = 80 cm^3^ and 220 cm^3^, respectively). The performance of SUVmax (AUC = 0.628; optimum cutoff = 17) did not reach statistical significance (*P* = 0.323). When cases were categorized using optimum cutoffs, higher SUVmax, MTV, and TLG were significantly associated with IS subtype (Table 3). To provide evidence that the use of two different PET/CT scanners did not likely influence our results, we performed additional subgroup analyses in subjects imaged with a STE PET/CT scanner (n = 79, 73.2%). The results were largely consistent with the main analysis findings (Supplementary Tables 1 and 2).

**Table 3:** Comparison of metabolic parameters according to immune subtype in the extra-nasal lesion subgroup.

	Immune subtype				
	IT (N = 5)	IE-A (N = 28)	IE-B (N = 14)	IS (N = 8)	P-value
**Metabolic parameters**					
SUVmax > 17	0 (0%)	8 (29%)	2 (14%)	5 (63%)	0.044†
MTV > 80	0 (0%)	3 (11%)	2 (14%)	5 (63%)	0.005†
TLG > 220	0 (0%)	11 (60%)	3 (21%)	7 (88%)	0.005†
**Site of SUVmax**					
Nasal	2 (40%)	14 (50%)	6 (43%)	1 (12%)	0.307†
Extra-nasal	3 (60%)	14 (50%)	8 (57%)	7 (88%)	
**Involved extranasal sites**					
Adrenal gland	0 (0%)	1 (4%)	3 (21%)	5 (63%)	0.001†
Testis	0 (0%)	1(4%)	2 (14%)	3 (38%)	0.043†
Gastrointestinal tract	2 (40%)	6 (21%)	3 (21%)	1 (13%)	0.710†
Lymph node	1 (20%)	15 (53%)	8 (57%)	4 (50%)	0.530†
Soft tissues	1 (20%)	8 (29%)	1 (7%)	2 (25%)	0.462†

IT, immune tolerance; IE, immune evasion; IS, immune silenced; SUV, maximum standardized uptake value; MTV, metabolic tumor volume; TLG, total lesion glycolysis; †, Chi-squared test.

### PET/CT pattern in the sub-population with extra-nasal FDG lesions

In this sub-population, the area of highest SUVmax was found in nasal FDG lesions in 42% of cases and in extra-nasal FDG lesions in 58% of cases. Extra-nasal FDG lesions were most frequent in lymph nodes, GI tract, and soft tissue (skin, subcutaneous, and muscle; Table 3). In the IS group, SUVmax was predominantly found in extra-nasal sites (88%), particularly in adrenal (63%, *P* < 0.001) and testicular FDG lesions (38%, *P* = 0.043).

### Proposed flow chart using FDG PET/CT to predict IS type

**Figure 2:**
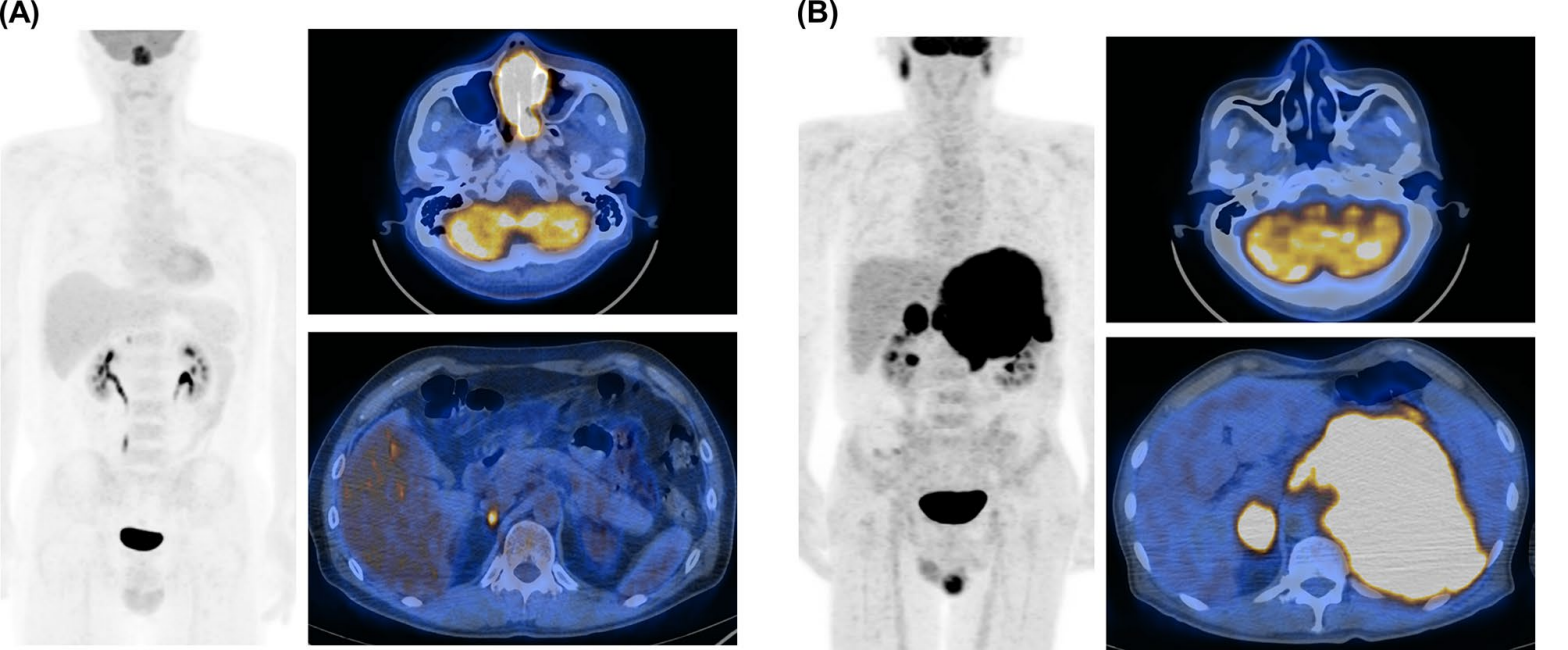
Representative PET/CT images of ENKTL patients. (**A**) Maximum intensity projection (left) and transaxial fusion PET/CT (right) images of a 50-year-old male showing tumor with highest SUVmax in the nasal cavity (MTV = 43, TLG = 318). The patient had a single IS-favoring PET pattern (small adrenal FDG lesion) and was confirmed to have IE-A subtype. (**B**) Maximum intensity projection (left) and transaxial fusion PET/CT (right) images of a 56-year-old male with tumor lesions in adrenal glands and testis. The patient had normal nasal cavity uptake but high MTV and TLG on adrenal lesions. This case had all four IS-favoring PET patterns and was confirmed to have IS subtype.

Finally, each case was classified based on FDG PET/CT pattern and volumetric parameters useful for discerning IS type. Representative PET/CT images of patients that displayed single and multiple IS-favoring findings are illustrated in Figs. 2A,B, respectively.

**Figure 3:**
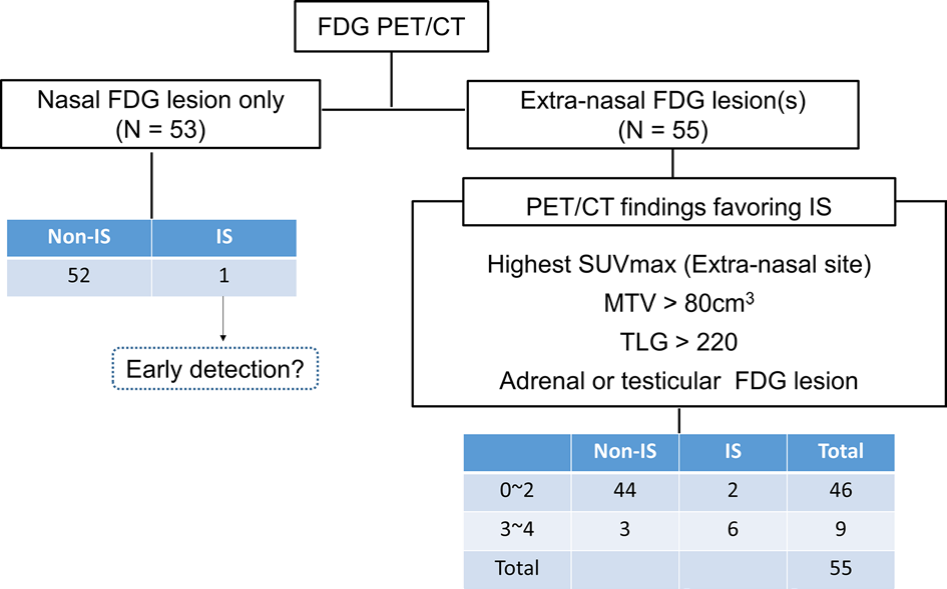
Flowchart using FDG PET/CT pattern and metabolic parameters to differentiate TIME subtype. Subjects with only nasal involvement had non-IS types in all but one case. In the extra-nasal lesion group, those with three or more favoring PET features had high probability of IS, whereas those with two or fewer features had low probability.

Analysis of all patients revealed that confinement of FDG lesions in the upper airway tract excluded non-IS type in 98% of cases (52/53; Fig. 3). There was a single case of IS type with FDG lesions only in the nasal area. It is possible that this represents a case detected at an early stage before dissemination had occurred.

The remaining 55 cases with extra-nasal FDG lesions were evaluated for PET/CT features that favor the IS type, including MTV > 80 cm^3^, TLG > 220 cm^3^, adrenal or testis involvement, and SUVmax located in extra-nasal sites. The presence of two or less of these four features correctly identified non-IS type in 44/46 cases (96%). Conversely, the presence of at three or all four of the features correctly identified IS in 6/9 cases (67%; Fig. 3).

### Prognostic value of IS-favoring PET/CT score

**Figure 4:**
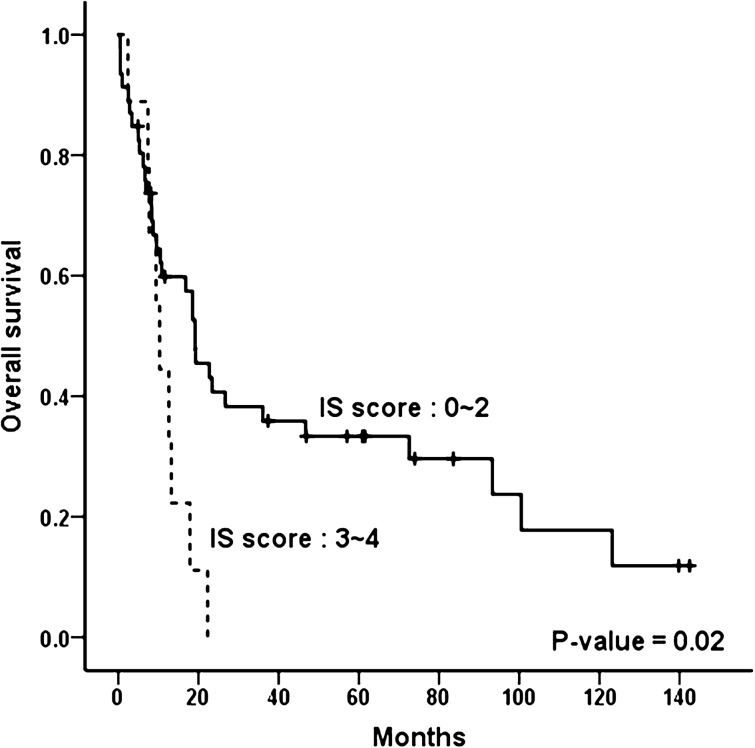
Patient outcome according to IS score. Overall survival was significantly inferior in patients who showed three or more IS-favoring PET/CT features compared to those who showed two or less such features.

When we compared the survival outcome according to IS score, overall survival of 55 cases with extra-nasal FDG lesions was significantly inferior in patients who showed three or more IS-favoring PET/CT features compared to those who showed two or less such features (*P* = 0.02; Fig. 4). In 37 patients with newly diagnosed ENKTL, low PET/CT scores (0–2) showed a trend for better overall (*P* = 0.14) and progression free survival (*P* = 0.34) compared to high scores (Supplementary Fig. 1. In this group, 34 subjects with non-IS types showed a similar trend of better overall (*P* = 0.12) and progression free survival (*P* = 0.22) when they had low PET/CT scores (Supplementary Fig. 2). When all subjects with IS subtype were assessed (n = 8), low PET/CT score (0–2) was observed to be associated with a positive impact on survival outcomes compared to high scores (Supplementary Fig. 3). However, statistical significance was not reached in the subgroup analysis, likely due to small subject numbers.

## Discussion

Progressive necrotic lesions, located mainly in the nasal cavity, are a major clinical feature of ENKTL that is associated with miserable prognosis and distant metastases^17,18^. Our results in this study of patients with locally advanced or disseminated ENKTL showed that pretreatment FDG PET/CT findings are significantly correlated with TIME subtype. In particular, patients with the IS subtype displayed FDG-avid lesions with higher SUVmax, MTV, and TLG, which were frequently present outside the upper respiratory tract, including adrenal glands and testis. These findings support the capacity of FDG PET/CT to help identify IS subtype, which we previously showed to have worse response to immunotherapy^4^. The relation between PET/CT findings and TIME subtype that we observed may be explained by the strong association reported between the metabolic status of tumors and their immune microenvironment^19,20^.

Dissemination of tumor cells from the primary site to distant organs is a complicated process that requires invasion, intravasation, circulation, colonization, and immune escape^11,12^. Lymphoma cells develop different mechanisms help adjust to the tumor microenvironment and foster their survival^21^. After leaving the primary origin, the cells choose microenvironments beneficial to their proliferation, differentiation and survival^22^. Our group previously categorized the TIME of ENKTL by immunohistochemical staining for CD68 (pan-macrophage marker) and PD-L1 (negative signal of effector T cells)^4^, where IT and IE subtype ENKTLs are surrounded by effector T cells. Such an immune microenvironment could contribute to strong FDG uptake due to infiltration of activated inflammatory cells^23^, and may serve as a metabolic barrier to the progression and dissemination of tumor cells^19,20^. Thus, our observation that IT and IE subtypes have strong FDG uptake in lesions restricted to the nasal cavity supports a dominant role of inflammatory cells in the immune environment surrounding ENKTL cells.

Conversely, IS subtype represents tumor cells encircling exhausted T cell activity. In patients showing this TIME subtype, lymphoma cells tended to have extranodal metastasis, including adrenal glands and testis, suggesting a lack of immunologic obstacles to pass through. This finding suggests that FDG intensity from extra-nasal lesions can reflect the metabolic activity of cancer cells, whereas non-lymphomatous inflammatory cells play an essential role in visualization of nasal lesions^24^.

Among FDG parameters, MTV and TLG displayed distinct differences according to TIME subtype. These volumetric parameters outperformed SUVmax values that showed considerable overlap between IE and IS groups. This indicates that volumetric metabolic tumor burden can better represent the TIME than SUVmax values obtained from a single voxel. This finding is consistent with recent reports that MTV and TLG were better predictors of prognosis in patients with ENKTL than was SUVmax^8,9^.

Despite the clinical usefulness of volumetric PET parameters, the best way of their measurement is not yet firmly established^25^. For DLBCL, MTV and TLG are generally measured from all FDG-avid lesions with a threshold of 41% maximum^26^. However, this method can potentially exclude large portions of lymphoma tissue with relatively low SUV^27^. Furthermore, a previous study showed that FDG volumetric data of different lymphoma sites are associated with divergent clinical significance^14^. ENKTL lesions have been shown to have metabolic activity lower than aggressive B-cell lymphomas, possibly due to large amounts of coagulative necrosis and inflammatory components in the tumor^28^. In addition, ENKTL is often disseminated to various organs, making it difficult to include all lesions with precision. For these reasons, we calculated MTV from a single lesion with the highest FDG intensity rather than attempt to measure the metabolic activity of the whole tumor burden.

Previous studies reported worse prognosis for primary testis and adrenal gland lymphoma following existing treatment strategies^29–31^. Our own recent research showed that IS subtype ENKTL responded poorly to the immune checkpoint inhibitors pembrolizumab and nivolumab under relapse settings^4^. When biopsy is unavailable, FDG PET/CT findings may indirectly help to differentiate IS subtype, allowing prediction of poor prognosis and low response to immune checkpoint inhibitors. Indeed, the results of the present study demonstrated that patients who showed more IS-favoring PET/CT features had worse overall survival compared to their counterparts.

When we finally stratified the probability of IS type using PET/CT features, the presence of only nasal FDG lesions (53 subjects) and two or less PET/CT features favoring IS type (46 patients) excluded IS type with a high negative predictive value (97.0%). Therefore, it might be considered likely that this group of ENKTL patients will show good response to immunotherapy. On the other hand, the presence of three or four IS type PET/CT features correctly identified the subtype with a positive predictive value of 66.7% (6/9). These results indicate that PET/CT feature offers a non-invasive method for predicting TIME subtype when biopsy is unavailable.

Furthermore, low PET/CT score was associated with more favorable survival outcome in patients with extra-nasal FDG lesions. It also showed a trend for association with better survival in newly diagnosed ENKL patients in both non-IS and IS subtypes. Therefore, PET/CT features favoring IS type may also be useful as prognostic indicator. However, since statistical significance was not reached in subgroup analysis, likely due to small subject numbers, this needs to be validated in larger cohorts.

Limitations of the study include the possibility of inherent biases associated with a retrospective study design as well as from a modest sample size. Imaging with one of two different PET/CT scanners in our patients is a potential source for variability in volumetric measurement. However, subgroup analyses in the subjects imaged with a STE scanner showed findings consistent with the main analysis. Taken together, although the rarity of the disease limits patient availability for clinical trials, our findings need to be validated by prospective studies with a larger sample size in a more homogeneous setting.

In conclusion, PET/CT pattern and volumetric FDG uptake parameters of extranodal lesions in patients with ENKTL helped distinguish IS from other TIME subtypes. This ability might assist in predicting response to immune checkpoint inhibitor therapies. We thus propose that FDG PET/CT may be helpful to discern immune subtype and forecast therapeutic response in patients with ENKTL.

## Supplementary Information

Supplementary Information.

## Data Availability

All data generated or analyzed during this study are included in this published article.
