# Supplementary material for: Metabolic activity of extranodal NK/T cell lymphoma on 18F-FDG PET/CT according to immune subtyping
Source: Sci Rep. 2021 Mar 15;11:5879. doi: 10.1038/s41598-021-85332-0 (PMC7960964; doi:10.1038/s41598-021-85332-0)

**Metabolic activity of extranodal NK/T cell lymphoma on ^18^F-FDG PET/CT according to immune subtyping**

**(Short Title:** FDG parameters and immune subtyping in ENKTL)

Chae Hong Lim^1†^, Sang Eun Yoon^2†^, Seok Jin Kim^2^, Junhun Cho^3^, Young Hyeh Ko^3^, Kyung-Han Lee^4*^, Won Seog Kim^2*^

^1^Department of Nuclear Medicine, Soonchunhyang University Hospital, Seoul, Korea

^2^Division of Hematology-Oncology, Department of Medicine, Samsung Medical Center, Sungkyunkwan University School of Medicine, Seoul, Korea

^3^Department of Pathology, Samsung Medical Center, Sungkyunkwan University School of Medicine, Seoul, Korea

^4^Department of Nuclear Medicine, Samsung Medical Center, Sungkyunkwan University School of Medicine, Korea

**Supplementary Table 1.** SUVmax according to immune subtypes (Subgroup analysis with STE scanner group; n = 79)

|  | **Immune subtype (Total)** | | | | | | | |
| --- | --- | --- | --- | --- | --- | --- | --- | --- |
|  | IT (N = 14) |  | IE-A (N = 43) |  | IE-B (N = 14) |  | IS (N = 8) | P-value |
| **SUVmax** |  |  |  |  |  |  |  |  |
| Median (25%~75%) | 8.7 (6.5-9.7) |  | 11.8 (8.8-17.1) |  | 9.7 (6.7-10.3) |  | 18.5 (10.8-21.0) | 0.011‡ |
| Mean ± SD | 9.3 ± 5.2 |  | 14.3 ± 8.8 |  | 8.9 ± 3.5 |  | 16.3 ± 6.7 |  |

**Supplementary Table 2.** Comparison of metabolic parameters according to immune subtype in the extra-nasal lesion subgroup (Subgroup analysis with STE scanner group; n = 39)

|  | **Immune subtype (Extra-nasal lesion subgroup)** | | | | |
| --- | --- | --- | --- | --- | --- |
|  | IT (N = 5) | IE-A (N = 20) | IE-B (N = 7) | IS (N = 7) | P-value |
| **Metabolic parameters** |  |  |  |  |  |
| SUVmax > 17 | 0 (0%) | 7 (35%) | 0 (0%) | 4 (57%) | 0.045† |
| MTV > 80 | 0 (0%) | 3 (15%) | 1 (14%) | 5 (71%) | 0.008† |
| TLG > 220 | 0 (0%) | 7 (35%) | 2 (29%) | 6 (86%) | 0.018† |

**Supplementary Figure 1.** Survival outcome according to IS-favoring PET/CT score in 37 patients with newly diagnosed ENKTL


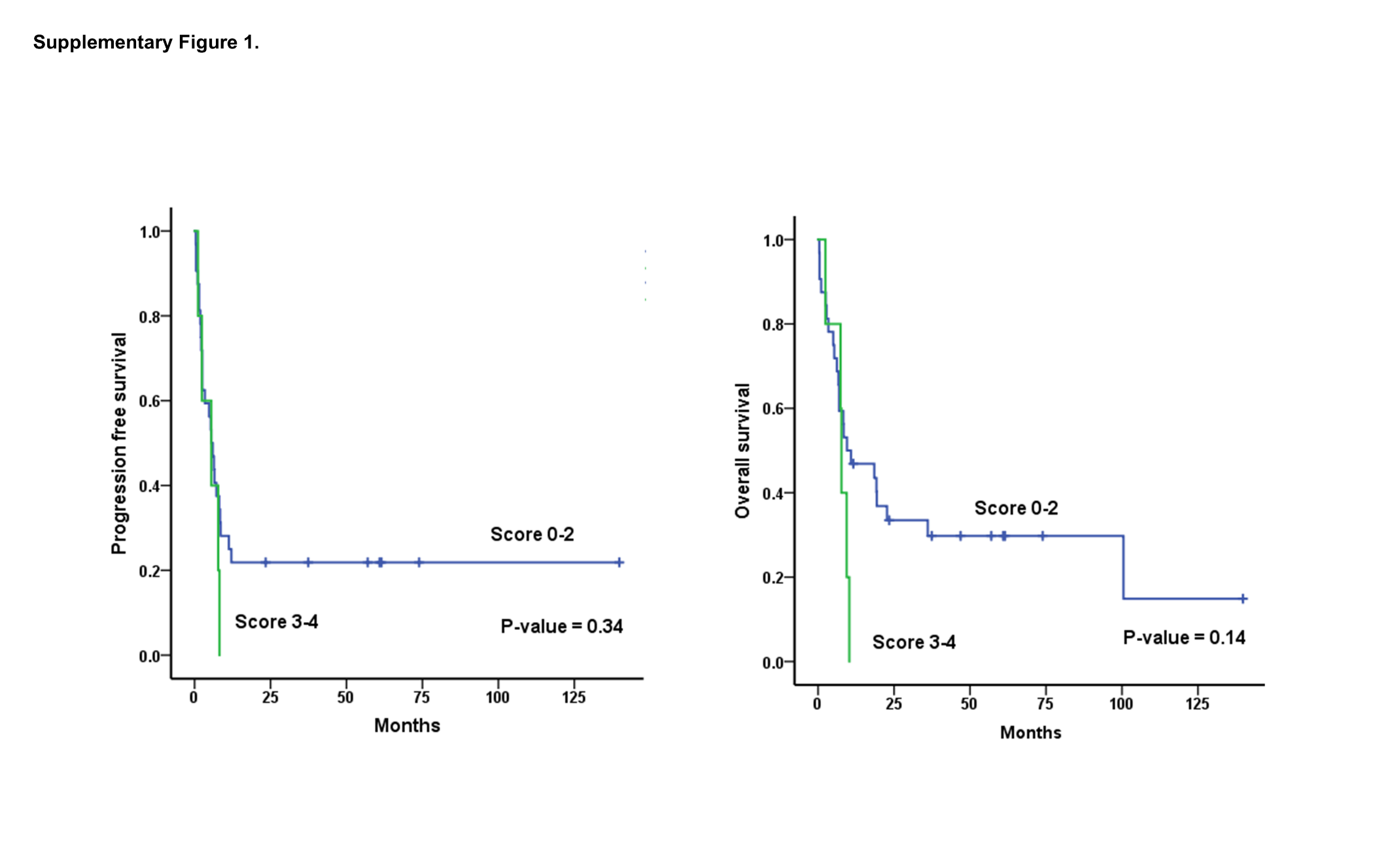


**Supplementary Figure 2.** Survival outcome according to IS-favoring PET/CT score in 34 patients with newly diagnosed ENKTL of non-IS subtype


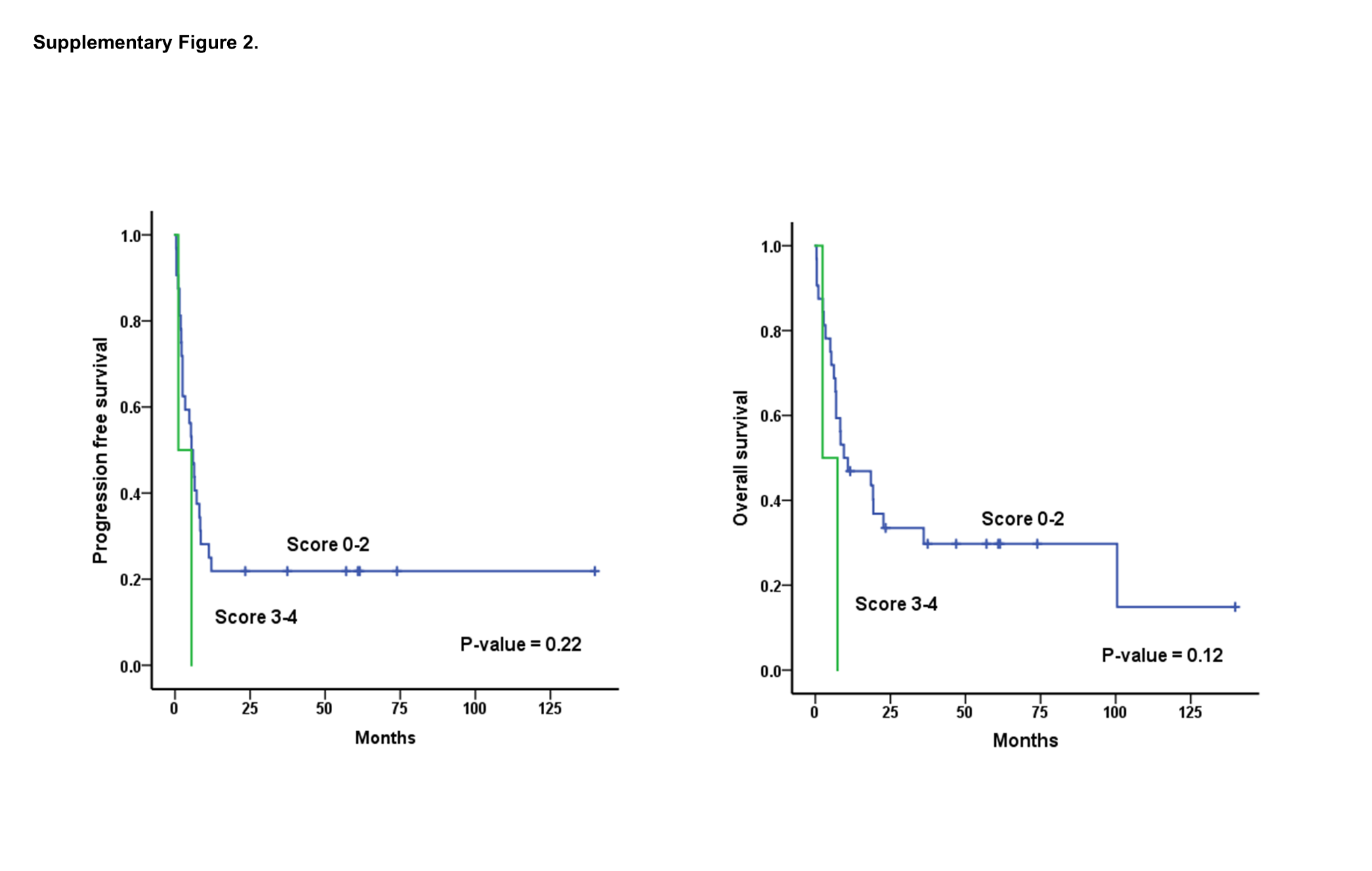


**Supplementary Figure 3.** Treatment and outcome according to IS-favoring PET/CT score in 8 patients with IS subtype


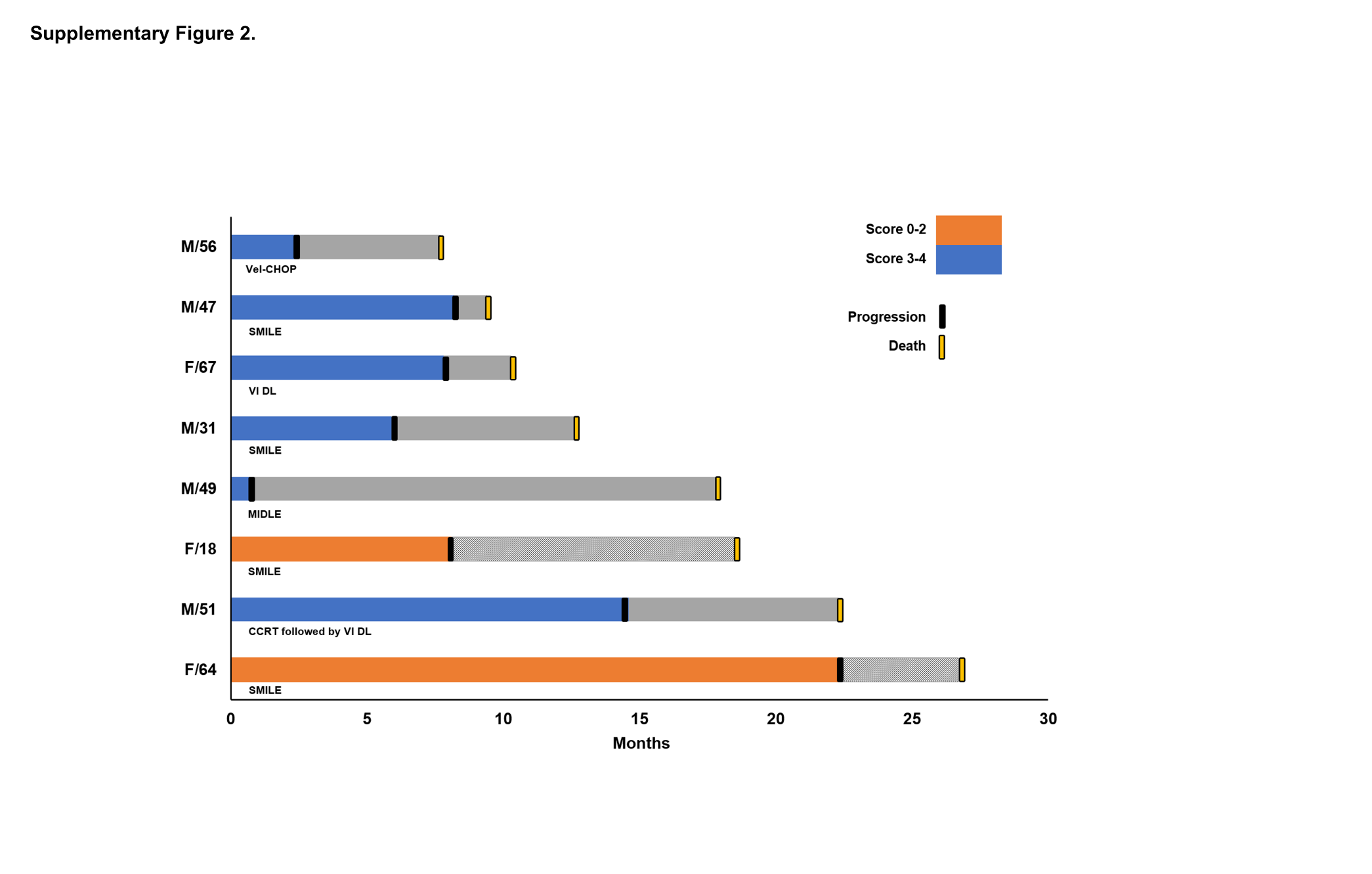

Supplement: Supplementary file 1 — Supplementary Information. [file 41598_2021_85332_MOESM1_ESM.docx]
